# An effective trap for spotted lanternfly egg masses

**DOI:** 10.3389/finsc.2023.1154510

**Published:** 2023-04-17

**Authors:** Phillip Lewis, Amanda Davila-Flores, Emily Wallis

**Affiliations:** Forest Pest Methods Laboratory, United States Department of Agriculture, Animal and Plant Health Inspection Service, Buzzards Bay, MA, United States

**Keywords:** spotted lanternfly, *Lycorma delicatula*, trapping, egg masses, *Ailanthus altissima*

## Abstract

Spotted lanternfly (SLF) (*Lycorma delicatula* (White)), an invasive planthopper discovered in Pennsylvania, USA in 2014, continues to spread and is now present in 14 states with substantial infestations present in seven states. Population projections using adult SLF trapping or visual counts are not reliable due to the transient, migratory behavior of the adults which make population forecasts difficult. Another approach to population monitoring is utilization of the stationary egg mass stage, but counting small cryptic egg masses throughout the canopy of large trees in dense woodlots is arduous and prone to error. After several field seasons testing various trapping configurations and materials, we have identified an efficient, simple, low-cost trap termed a ‘lamp shade trap’ that is attached to the lower trunk area of an SLF host tree. SLF females readily enter the trap and lay eggs on the thin, flexible trap surface. A vertical trap orientation was superior, and the most productive woodlots yielded an average of 47 and 54 egg masses per trap, and several traps had over 100 egg masses. There were 1,943 egg masses tallied from 105 traps placed at six locations in two states. Egg mass counts in the area above and below the traps and on nearby control trees yielded very few egg masses in comparison. Selection of trees 15 to 20 cm in diameter for trap placement is most efficient, yielding good egg mass abundance while minimizing the amount of trap material used. The lamp shade trap has potential as an effective tool to identify SLF in new areas, gauge SLF population levels in woodlots and can also be used to collect and monitor egg masses for research purposes.

## 1 Introduction

The spotted lanternfly (SLF), *Lycorma delicatula* (White) (Hemiptera: Fulgoridae), is an invasive, destructive fulgorid that has gained a strong foothold in the eastern United States since it was first discovered in Pennsylvania in 2014 (1). In a short period of time this sap feeding insect native to Taiwan and China (2) has greatly expanded its range and there are now portions of 14 states with established populations as well as detections of SLF in two additional states (3). This insect is typically a pest of the invasive tree of heaven (TOH), *Ailanthus altissima* (Miller) Swingle (Sapindales: Simaroubaceae) but feeds on a broad variety of host plants and trees (2, 4, 5). In the state of Pennsylvania, it has and continues to cause devastation to a number of commodities and industries including grape, forest timber and ornamental tree production (6–8).

The potential for this pest insect to expand its population is heightened by the young nymphs (1^st^ to 2^nd^ instars) which are very active and polyphagous and remain widely dispersed as they mature on a large variety of plants (4). The 4^th^ instar (red form) and newly molted adults begin to congregate in large numbers on TOH and other preferred host trees where heavy feeding commences for several weeks before adult courtship and mating activities. During the mating period there are aggressive adult migration and dispersal events (9). Due to their extreme mobility the prediction and estimation of nymph and adult SLF population levels is confounded by an insect which does not actively seek out traps due to the lack of an effective lure (10). SLF aggregation behaviors are not well understood and may be driven by a combination of changing nutritional levels in their host plants as well as for their own needs (11) making it almost impossible to estimate and predict population levels.

Another approach to predicting SLF populations is monitoring of the overwintering egg stage. SLF typically lays eggs from September until early December and egg masses can contain 30 to 50 eggs covered in a yellow-brown waxy covering (12). Egg masses are laid mostly on the bark of trees but can be found on almost any flat surface including vehicles, stones, fence posts, buildings and backyard play equipment (1, 13). Location of egg masses can vary within a tree and are correlated to tree height. For example it was found that egg masses on TOH were concentrated towards the lower 2.5 m of the tree in areas where trees were < 6 m in height (14). Another study (15) found egg masses concentrated above 6 m on TOH when tree height ranged from 5.5 - 23.8 m. SLF egg mass survey activities are common during winter months and have been used to assess populations and gauge infestation levels (15), however the cryptic nature of SLF egg masses and the fact that eggs are most likely higher up in the canopy make visual egg mass surveys unreliable.

Observations by the authors of SLF egg mass locations (e.g. underside of limbs, on fence posts, play equipment, wheel wells of vehicles and on a rusty lid from 55-gallon drum) and that masses were sometimes clustered, indicated to us that perhaps oviposition behavior was not completely random and could be directed. In the fall of 2018, we initiated investigations to see if SLF females had oviposition preferences that would induce them to lay their eggs on a trap or removable surface. In subsequent years we continued to test materials and environments and made incremental progress in identifying what worked and what didn’t. We noted certain preferences in materials or eliminated trap designs or approaches that female SLF failed to interact with. In 2022 we settled on a single trap design that combined two key attributes that we were able to identify: a preferred material for egg laying and a protected area/environment that we hoped would induce egg laying behaviors.

Trap appearance is that of a lamp shade and the traps are very efficient at concentrating SLF egg masses. Very few egg masses were noted above and below the traps or on nearby trees. These lamp shade traps provide an environment and a substrate on which SLF females readily oviposit. The traps have potential to be a valuable research tool not only to collect and monitor SLF egg masses but potentially for detecting SLF in new areas, for monitoring SLF in areas of concern and potentially for estimating and predicting SLF population levels in infested areas and woodlots.

## 2 Materials and methods

### 2.1 Study sites

**Figure 1:**
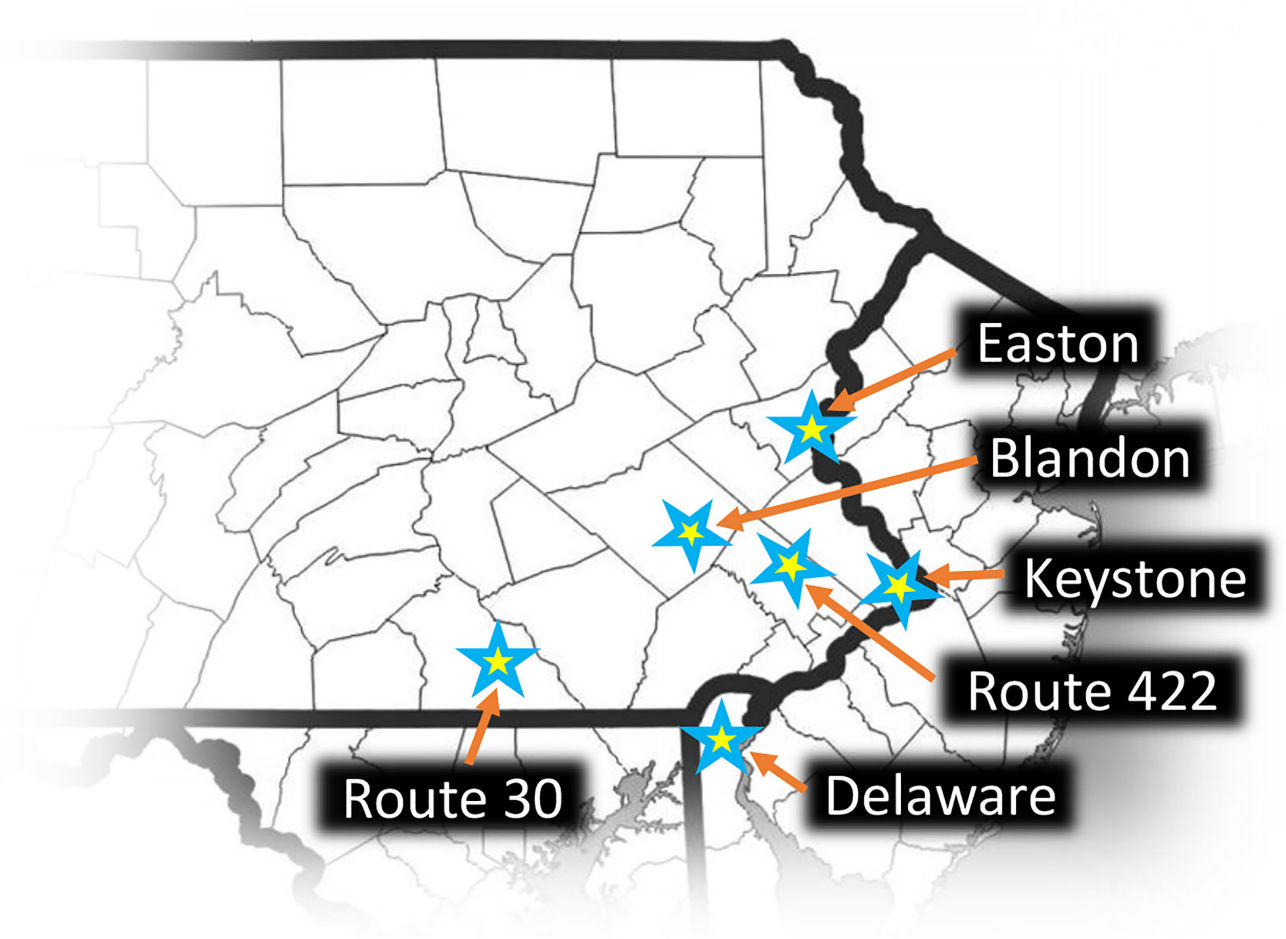
Study sites where lamp shade egg mass traps were deployed. Map used by permission from www.amaps.com.

Traps were deployed on TOH at locations where adult SLF populations were known to be medium to high. All sites were dominated (>80%) with mature TOH of various diameters with very little understory. Six sites were used from six different counties in southeastern Pennsylvania and in northern Delaware. Site locations are noted in **Figure 1**. Additional location information and the name designations used in this report were as follows: Blandon (Blandon, PA; Berks Co.; 40.442, -75.880); Route 422 (Royersford, PA; Montgomery Co.; 40.172, -75.504); Easton (Easton, PA; Northampton Co.; 40.678, -75.194); Delaware (New Castle, DE; New Castle Co.; 39.710, -75.568); Keystone (Fairless Hills, PA; Bucks Co.; 40.170, -74.753); Route 30 (Wrightsville, PA; York Co.; 40.029, -76.550).

### 2.2 Trapping approach 2018-2021

**Figure 2:**
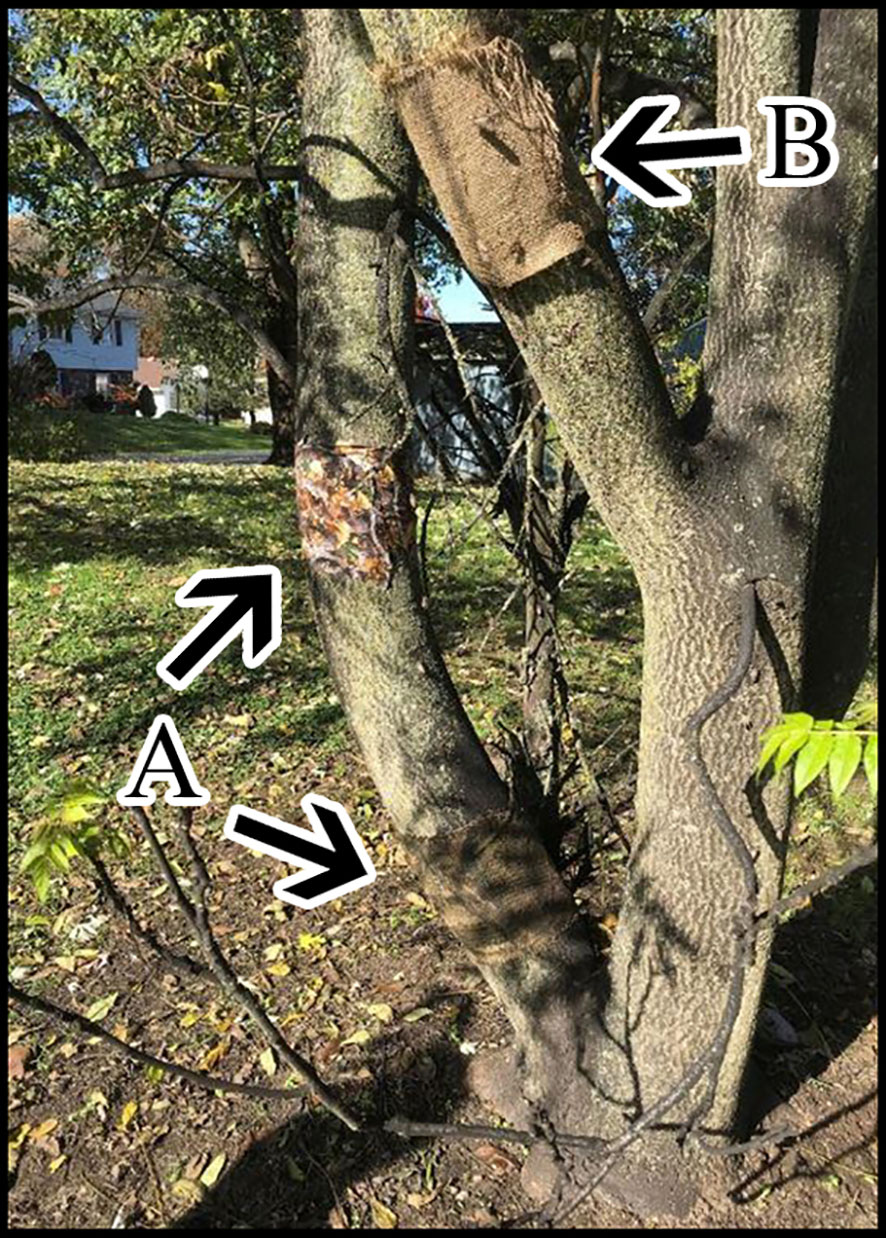
Fabric pieces arranged on a TOH as indicated by arrows, **(A)** camo fabric wrapped around the trunk at 1.4 m and burlap fabric wrapped around the base of the tree, **(B)** hanging burlap fabric stapled along the top edge.

Initial trapping designs in 2018 consisted of 30 x 50 cm burlap or cotton fabric materials and an artificial bark product (PINVNBY, available from Amazon Inc.). Materials were stapled directly to TOH trunks and a few other tree species at diameter breast height (DBH; 1.4 m) and at the base of the trees. Attachment was flush (**Figure 2A**) or the material was stapled at the top allowing it to hang off the trunk (**Figure 2B**). We placed a total of 75 objects at three locations where SLF adults were active.

**Figure 3:**
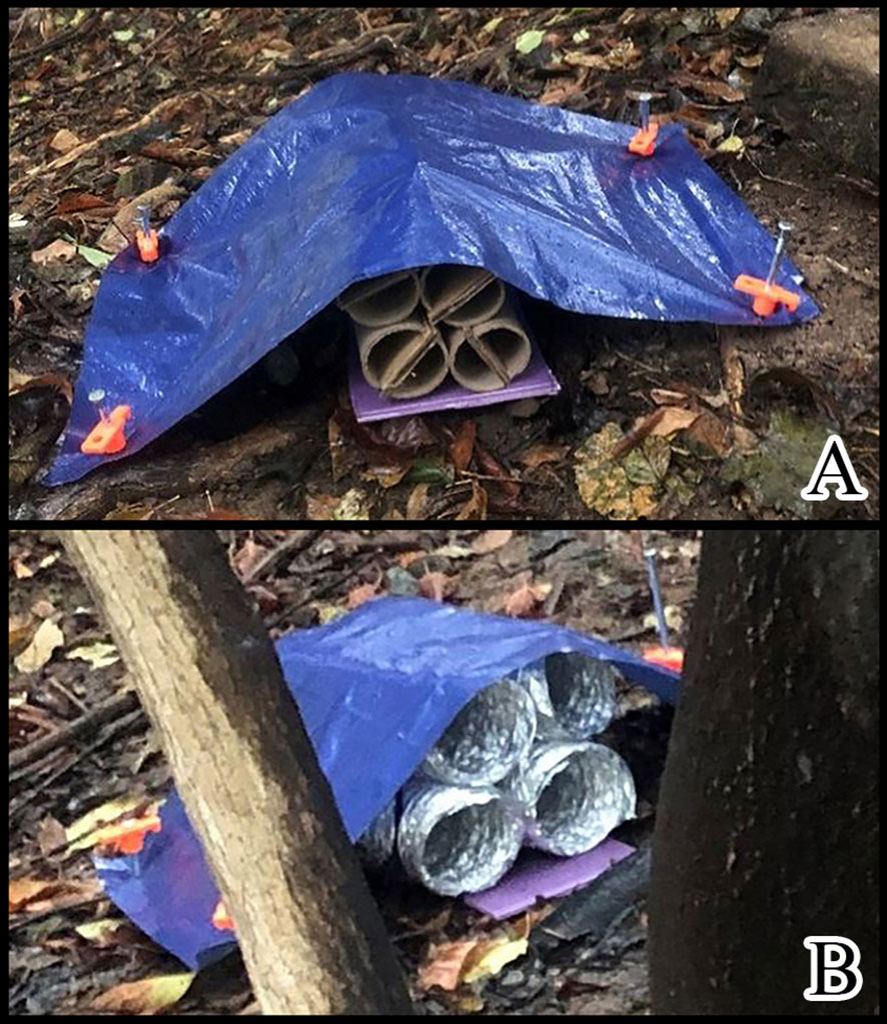
Tube hotels placed at ground level next to the base of a TOH, **(A)** cardboard tubes, **(B)** metal duct tubes.

**Figure 4:**
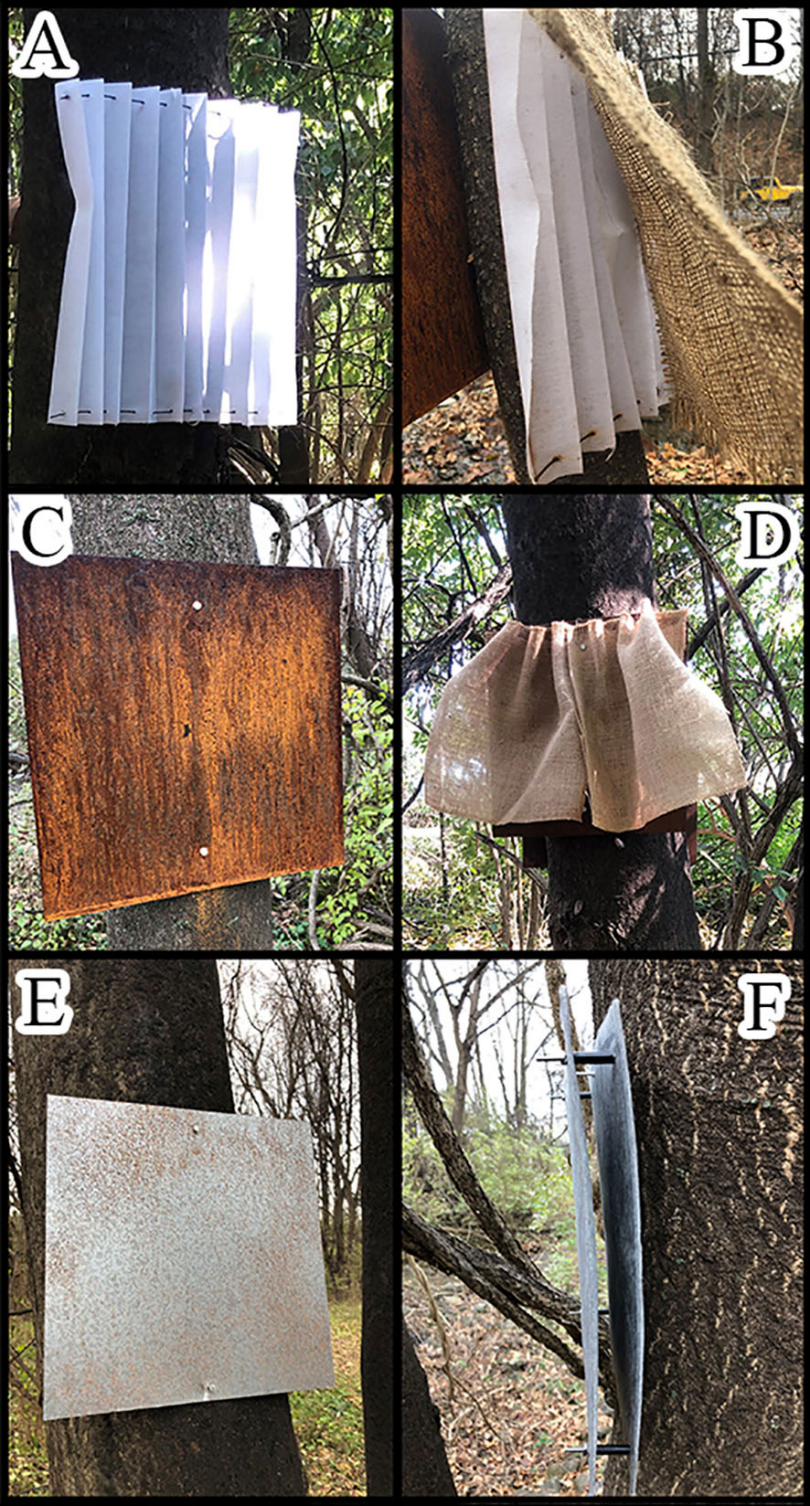
Set of cellulose traps, **(A)** without burlap, **(B)** with burlap, **(C)** rusty metal without burlap, **(D)** rusty metal with burlap, **(E)** metal with Rust-Oleum^®^ spray, **(F)** metal sandwich wrapped with landscaping fabric with spacers in between.

**Figure 5:**
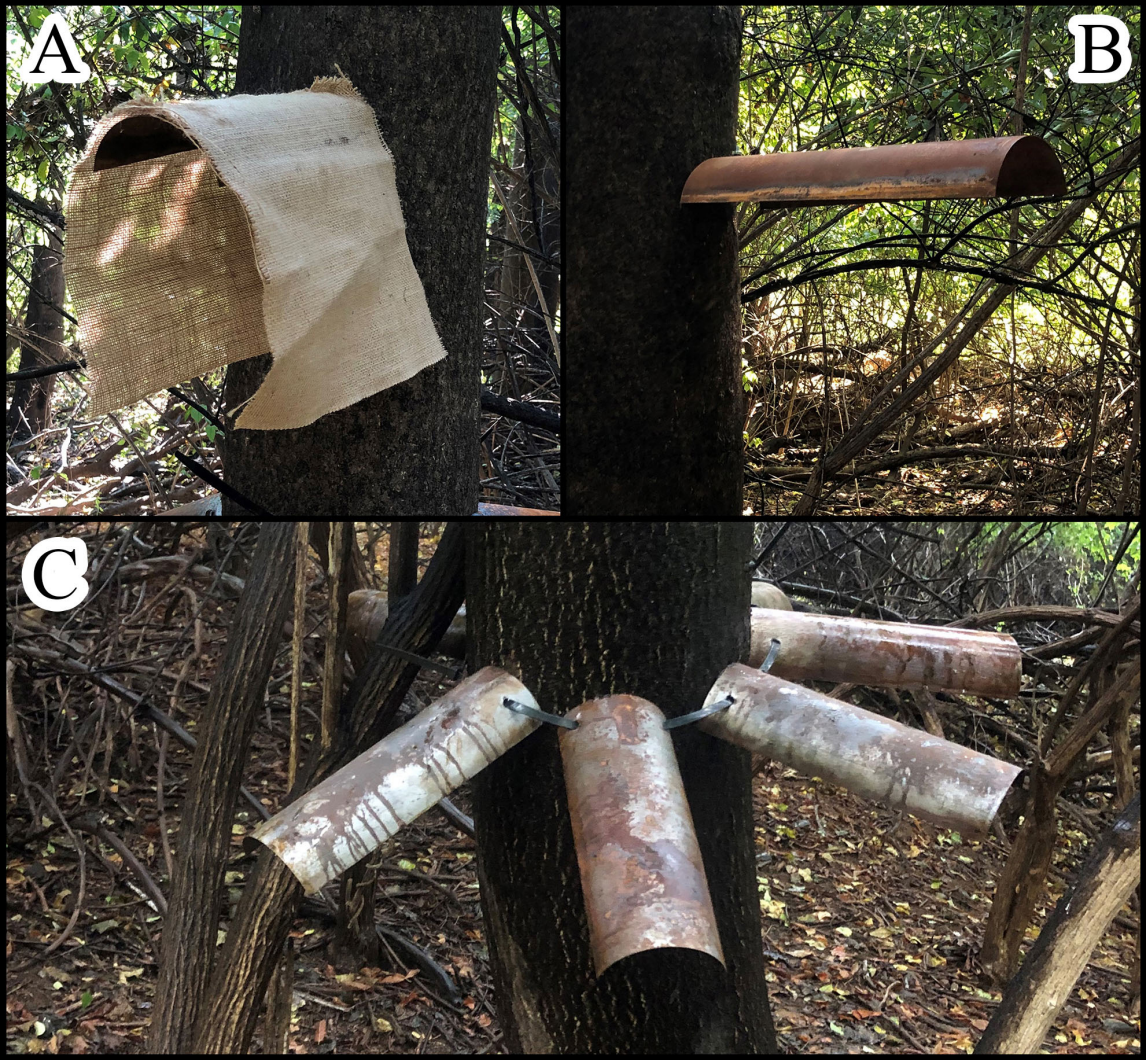
Half pipe rusty metal traps, **(A)** with burlap, **(B)** without burlap, **(C)** starfish formation around the trunk of the tree.

Trapping designs in 2019 included ground traps at the base of TOH and a wide variety of designs that were attached to TOH at DBH. The ground traps consisted of bundles of four 0.3 m long by 0.1 m diameter cardboard or aluminum tubes (dryer ducting, General Electric, Boston, MA), held together by zip ties with a piece of 6 mm thick tarp staked over to keep it secure to the ground to protect from the elements (**Figures 3A, B**). Traps attached to TOH included a 30 x 30 cm sheet of either cellulose shade fabric framed with wire (**Figures 4A, B**), rusty metal (**Figures 4C, D**), or metal with Rust-Oleum^®^ stone texture (Vernon Hills, IL) sprayed on (**Figure 4E**). These were placed as pairs either with a 30 x 30 cm piece of burlap hanging or the trap alone. Another set of traps used two pieces of 18-gauge stainless steel wrapped with landscaping fabric and held together with spacers to create a sandwich (**Figure 4F**). Traps that incorporated rust used 18-gauge galvanized metal that had been sandblasted and then sprayed with an oxidizing solution (32:8:1 mixture of hydrogen peroxide:vinegar:salt). Rusty metal traps consisted of a series of 15 x 30 cm half pipe pieces attached to the tree at DBH either alone (**Figure 5A**) or with a piece of burlap hanging over it (**Figure 5B**). A final metal trap was in a “starfish” configuration that encompassed the tree trunk but had no burlap (**Figure 5C**). We placed a total of 200 traps using 11 configurations and evenly distributed them at eight study sites. Traps with and without burlap included half pipe and sheet metal (24 each). There were two types of tube hotels and sandwich traps (24 each) and single traps of the following were placed at each site: metal sprayed with Rust-Oleum^®^, shade, shade with burlap and starfish.

**Figure 6:**
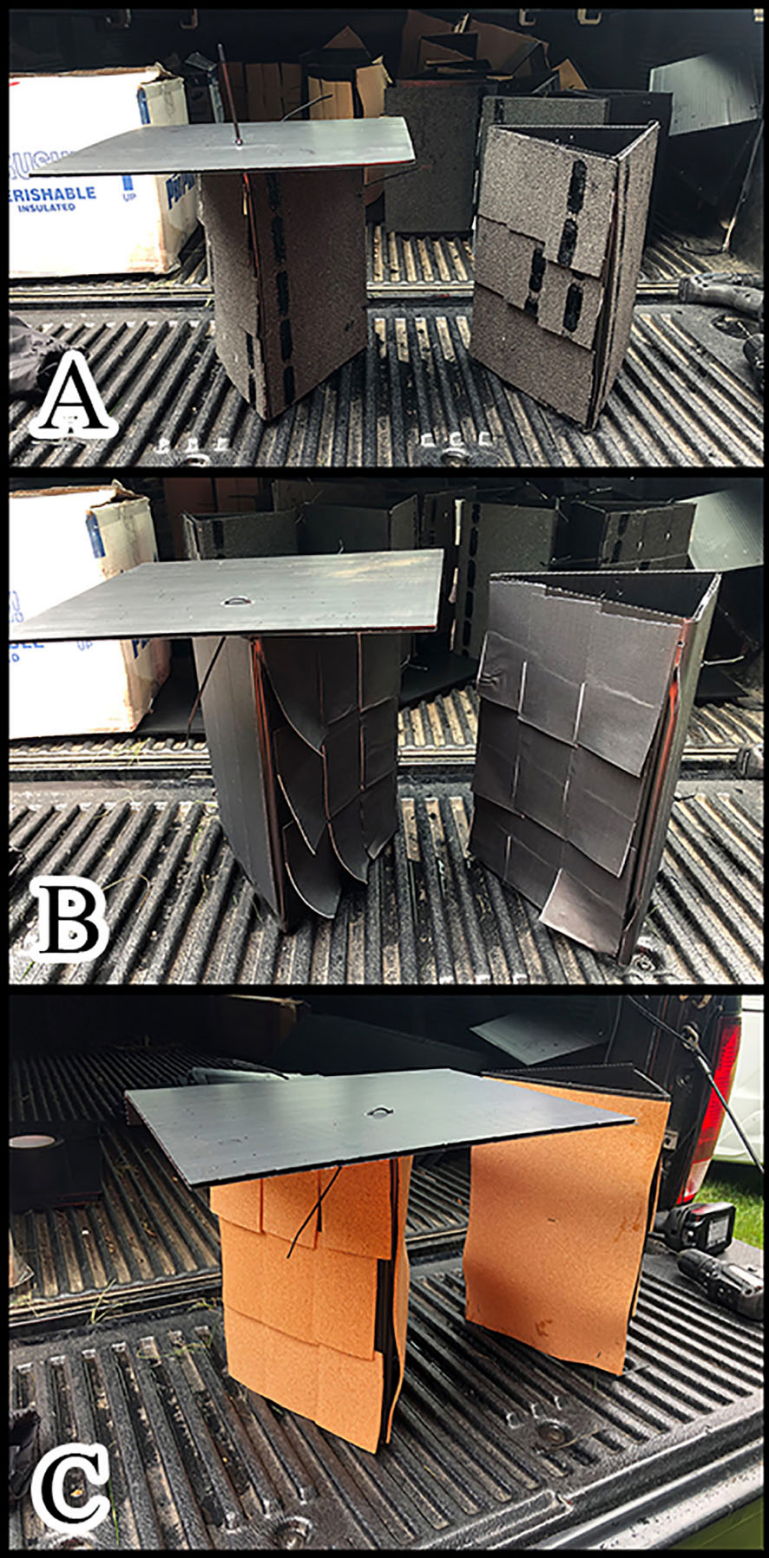
Triangle traps, **(A)** covered and uncovered with roofing, **(B)** covered and uncovered with gaffing tape, **(C)** covered and uncovered with cork. Each trap had one side with slightly overlapping strips of material and another side with a single smooth surface.

In 2020 we used a common design and focused on finding suitable oviposition materials. We constructed triangular traps out of 30 x 60 cm panels of black corrugated plastic (Uline, Pleasant Prairie, WI). One side was attached to the tree with staples or zip ties and the two outward facing surfaces were affixed with either ½” cork (Natural cork; Manton Cork, Hauppauge, NY), roofing material (Quick Start shingle roll; GAF, Parsippany, NJ) or gaffing tape (Lockport, Inc., Great Neck, NY). Materials were placed on the panels as either a single layer or using three over-hanging horizontal strips (**Figures 6A-C**). Each of these trap types was paired with an identical trap that was covered on the top with a 30 x 30 cm piece of the corrugated plastic. Traps were placed at a height of 3 m, 1.4 m and at the base of the tree. There were 156 traps placed at three study sites with eight or nine traps in each group.

**Figure 7:**
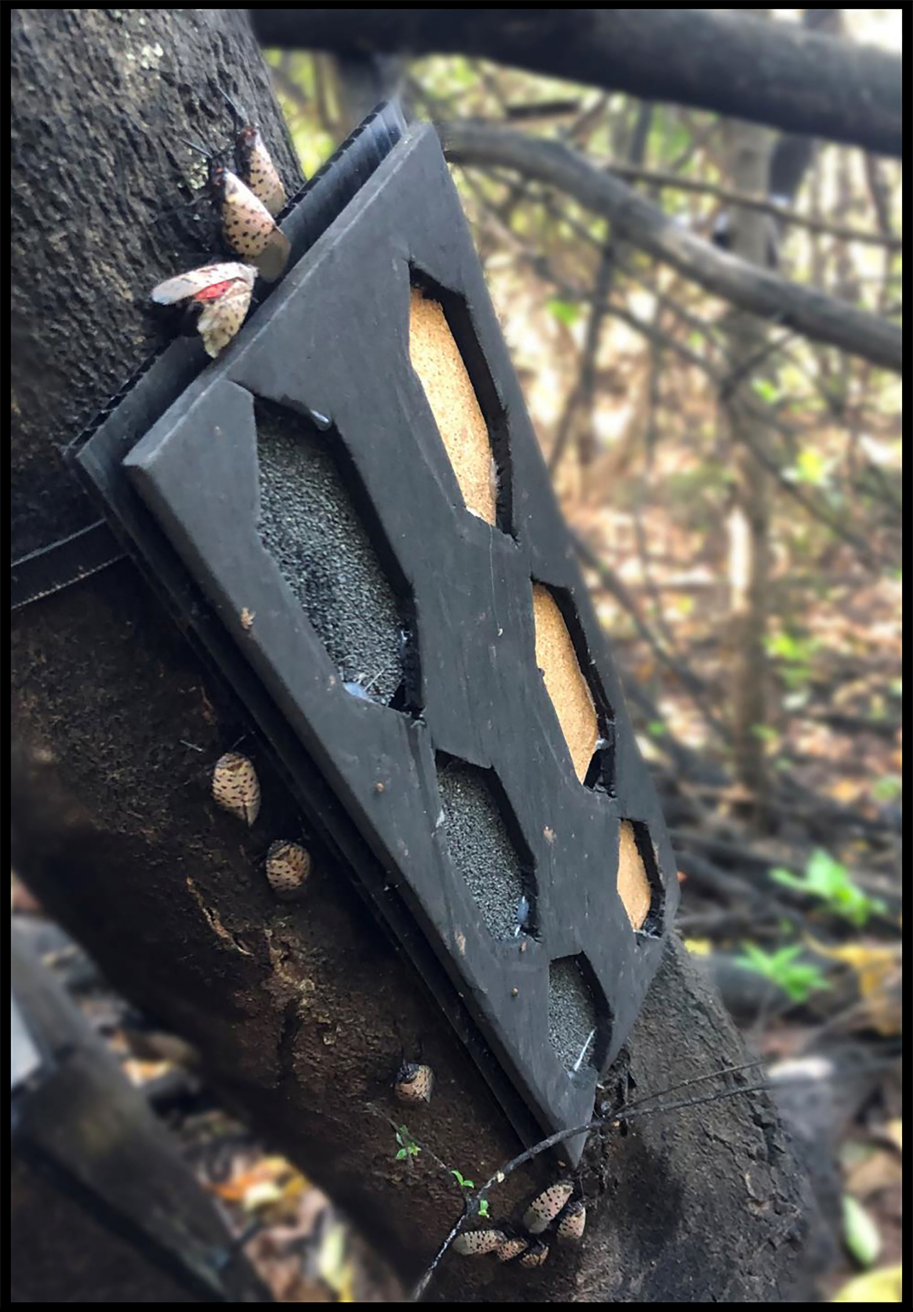
Hexagonal trap with roofing material and cork as egg laying substrates attached to *A. altissima*.

Trapping designs for 2021 used the same triangular traps but the oviposition substrate consisted of a single layer of roofing or cork material applied to the inside surface of the panels. Traps were tested at 1.4 m and at the base of TOH and had covered and uncovered pairings as in the previous year. There was a second trap type tested that used a 30 x 60 cm panel of corrugated plastic overlaid with a second panel that had six large hexagons cut into it, backed with cork and roofing material (**Figure 7**). We deployed 144 traps at three study sites. There were 3 traps tested of each type (2 materials covered or not, hexagon) at two heights for each site.

### 2.3 Trapping approach 2022

**Figure 8:**
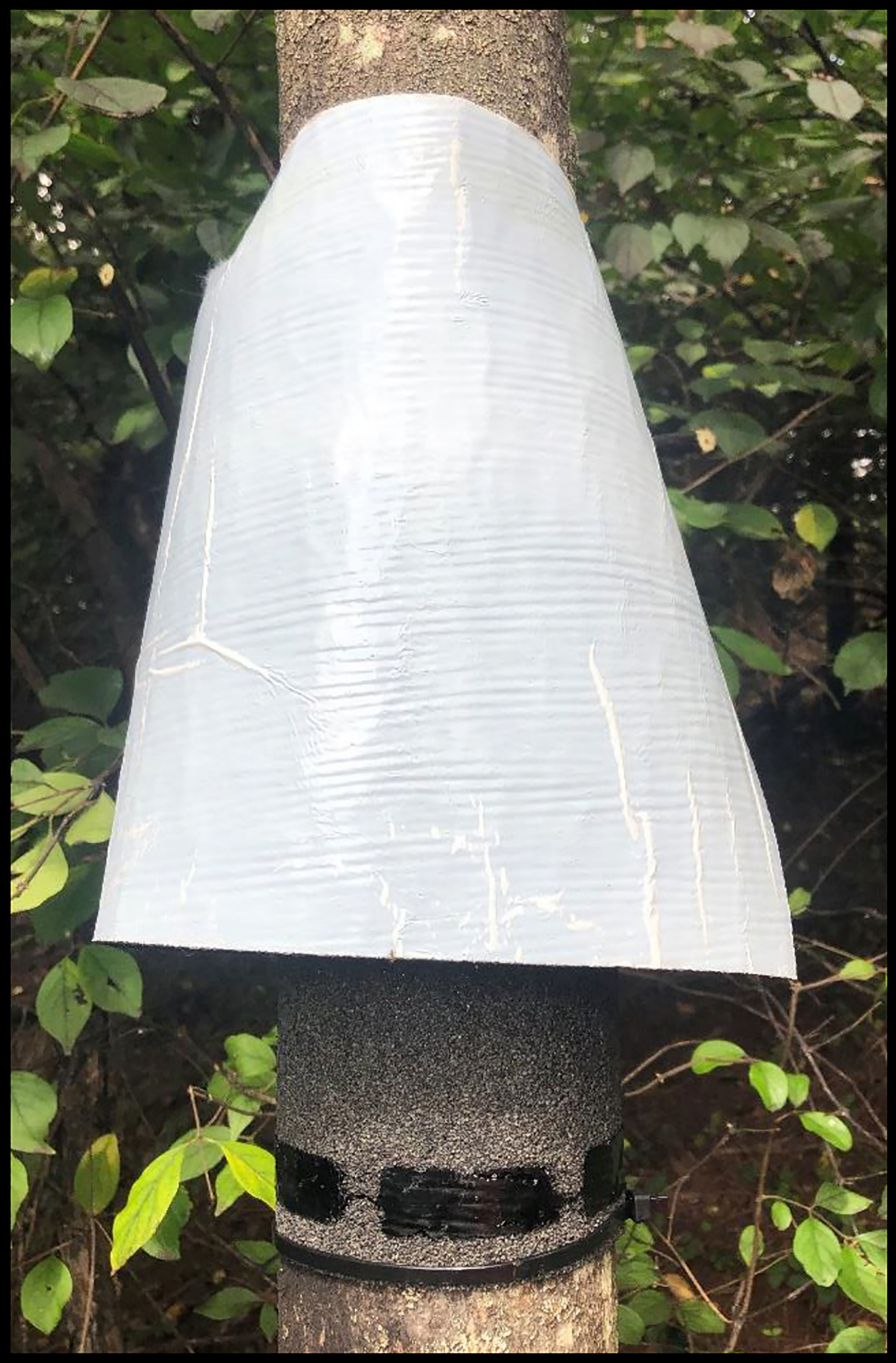
Lamp shade egg mass trap constructed on *A. altissima*.

The final trap design tested only roofing material as an oviposition substrate. In previous trap designs SLF females had to move off the tree trunk to encounter the substrate. This time we affixed the roofing material directly on TOH by wrapping it around the trunk at a 1.4 m height and we selected a range of tree diameters (± SE) for both vertical (Ave = 19.7 ± 0.8 cm; range = 9.7 to 35.1 cm; n = 73) and horizontal trap orientations (Ave = 11.4 ± 0.8 cm; range = 7.6 to 22.9 cm; n = 32). The trap material is overlapped slightly and stapled, and a zip tie is used to cinch the lower portion to the tree. At the top, batting material (9 cm wide, 2.5 cm thick) is attached, folded in half, and secured with a zip tie. The batting prevents SLF from passing through the trap while also holding the second layer of the trap away from the tree, creating a gap and a lamp shade appearance. The second layer of roofing material is inverted, and the top edge stapled to the tree right above the ring of batting fiber. The roofing material is positioned such that the asphalt sides of the two layers face each other with enough space between them to provide a protected area for the SLF females to interact with the trap substrate. **Figure 8** provides a picture of a lamp shade trap (LST). Detailed instructions, step by step pictures during construction and a supply list is available in the supplemental materials (pdf file name: LST_Construction).

In the fall of 2022 we deployed 105 LSTs on TOH at infested sites at six locations in multiple counties in southeastern Pennsylvania and in northern Delaware (**Figure 1**). Installation of the traps began 21 September (Blandon) and two additional trap sites were set up in the two weeks that followed (Route 422 and Delaware). The other three sites were set up the week of 16 October. Trap removal and assessments were done between early December of 2022 and early January of 2023, well after SLF oviposition had ceased. SLF egg masses laid on the traps were counted during trap take down and additional egg masses were noted that were present on the trunk above and below the trap to a height of 3 m. Additionally a nearby TOH control tree of similar diameter to each trap tree was selected (n = 105) and all SLF egg masses on that tree trunk were counted to a height of 3 m.

### 2.4 Data analysis

Statistical comparisons were not conducted in 2018 to 2020 due to the small numbers of egg masses laid on traps not allowing for robust statistical comparisons. Trapping data from the 2021 and 2022 field seasons were analyzed using Statistix 10 software (Analytical Software, Tallahassee, FL). The data from the six study sites were not normally distributed and data were not normalized by transformation. Trapping data were not grouped across sites but were analyzed independently for each study site. Egg mass data comparisons for where oviposition occurred (trap, above and below the trap, control tree) were analyzed for each site with a Kruskal-Wallis one-way ANOVA with an *a priori* significance level of α = 0.05. A similar approach was used for the comparisons of the area surveyed (m^2^) of where oviposition occurred.

For each study site, Wilcoxon Rank Sum tests were used to assess differences between the number of egg masses laid due to the vertical or horizontal orientation of the traps. Variance in the data for the DBH groupings were not normally distributed and raw data were used to perform a one-way ANOVA for comparisons of both the egg mass counts and the trapping surface area comparisons.

## 3 Results

### 3.1 Trapping results 2018-2021

Our initial approach in 2018 using fake bark, burlap and fabric materials resulted in 34 total egg masses on the 75 objects deployed and no material or configuration was preferred for oviposition. During assessments we did note that one horizontal tree trunk draped with burlap had around a dozen egg masses laid in a row where the fabric and tree bark intersected. We also came across a single rusty metal lid leaning against a tree that had 25 egg masses on the protected side. The following year we set out a total of 200 objects, many of them focusing on rusty metal as well as various materials draped over the traps. These were set up at eight sites but when traps were checked only 31 egg masses had been laid on the traps in a random manner with no noted preferences.

Trapping in 2020 focused on suitable oviposition materials and height placement of the traps. There were 156 triangle traps set up at three study sites. However, only seven egg masses were observed on the trap surfaces. Notably, all egg masses were laid on traps that had a top or cover placed on them, but trap catch was so low that robust statistical comparisons could not be performed. In 2021 the same triangle traps were used but oviposition substrate was positioned on the interior portion of the traps. Covered and uncovered traps were paired and placed at the base of the trees and at DBH. Of the 144 traps deployed there were 326 egg masses, most of which were laid on traps that were covered. One site had only two egg masses laid on the traps and was excluded from the analysis. Trapping data for the remaining sites were pooled and a Wilcoxon Rank Sum test showed a significant difference. Covered traps averaged (± SE) 5.5 ± 1.4 egg masses and traps without covers averaged 1.3 ± 0.4 (W = 2.20, *P* = 0.028), a four-fold increase. Trap height and substrate material comparisons were not significant (W = 0.44, *P* = 0.66 and W = 1.16, *P* = 0.25).

### 3.2 Trapping results in 2022

The 105 LSTs deployed in 2022 were very attractive to SLF females for oviposition and 1,943 egg masses were laid upon the trap surfaces. The vertical orientation of the trap was highly preferred for egg laying. High variance in the pooled data across study sites did not allow a combined analysis and trapping parameters (tree DBH, trap orientation and egg mass location) were analyzed independently for each study site. Numbers of vertical traps deployed, tree DBH information, average number of egg masses and average egg masses calculated by surface area on the vertical traps, masses observed above and below vertical traps as well as on control trees from the base to 3 m are given in **Table 1**, summarized by study site. The average number of egg masses laid on vertical traps varied from 9.6 to 54.4 masses for the six sites with a mean value of 25.4 ± 2.9 (SE) egg masses per trap and three individual traps captured 98, 102 and 111 SLF egg masses. The average number of egg masses laid in the traps was compared at each site with the number of masses above and below the traps. Egg masses from the base up to 3 m on control trees of similar size without a trap were also recorded and compared with trap tree data. All Kruskal-Wallis ANOVA comparisons were significantly different (*P* < 0.001), with the traps being highly preferred for oviposition by SLF females (**Table 1**). This preference is even more pronounced when the surface area of each trap is calculated and compared to the surface areas surveyed for SLF egg masses present above and below the traps and the egg masses present within the survey area of the control trees (last three columns, **Table 1**). Average number of egg masses by trap area (m^2^) ranged from 0.203 to 1.080 while egg masses found adjacent to the traps and on control trees averaged far fewer, between 0.0 to 0.023 egg masses per m^2^.

**Table 1:** Trapping information and abundance of SLF egg masses found on and around vertically oriented traps, by site.

Study Site	No. Traps Deployed/Date	Ave. DBH in cm (range)	Ave. No. Egg Masses per Trap	Ave. No. Egg Masses Above/Below	Ave. No. Egg Masses Control Tree	Ave. Egg Masses/m^2^ of Trap Area	Ave. Egg Masses/m^2^ Above/Below	Ave. Egg Masses/m^2^ Control Tree
Blandon	8; 9/21	18.5 ± 3.8 (9.7-33.0)	28.63 ± 6.05a	0.75 ± 0.37b	0.38 ± 0.18b	0.667 ± 0.095a	0.003 ± 0.001b	0.019 ± 0.001b
Route 422	8; 9/28	20.8 ± 3.8 (10.2-35.1)	54.38 ± 12.70a	5.38 ± 3.31b	8.50 ± 5.81b	1.080 ± 0.288a	0.019 ± 0.008b	0.023 ± 0.013b
Easton	8; 10/20	17.3 ± 2.0 (9.9-28.2)	9.63 ± 3.12a	0.00 ± 0.00b	0.25 ± 0.25b	0.203 ± 0.059a	0.0 ± 0.0b	0.001 ± 0.001b
Delaware	8; 10/5	19.6 ± 0.8 (17.3-22.6)	47.13 ± 12.11a	0.63 ± 0.26b	1.00 ± 0.46b	0.957 ± 0.276a	0.003 ± 0.001b	0.004 ± 0.019b
Keystone	20; 10/18	20.1 ± 1.8 (10.9-33.0)	14.45 ± 1.92a	5.20 ± 1.69b	2.25 ± 0.79b	0.296 ± 0.041a	0.018 ± 0.005b	0.007 ± 0.002b
Route 30	21; 10/19	19.1 ± 1.0 (11.7-25.4)	21.38 ± 3.86a	4.81 ± 1.45b	4.57 ± 1.11b	0.426 ± 0.068a	0.023 ± 0.008b	0.019 ± 0.005b

Egg mass counts were tabulated for each trap, above and below the traps up to a 3 m height and from the trunk of a nearby tree that had no trap up to a 3 m height. Average values are followed by the standard error; different letters denote statistical significance (P < 0.001; Kruskal-Wallis ANOVA) at each site and for each data grouping (average egg masses and average masses by area).

**Figure 9:**
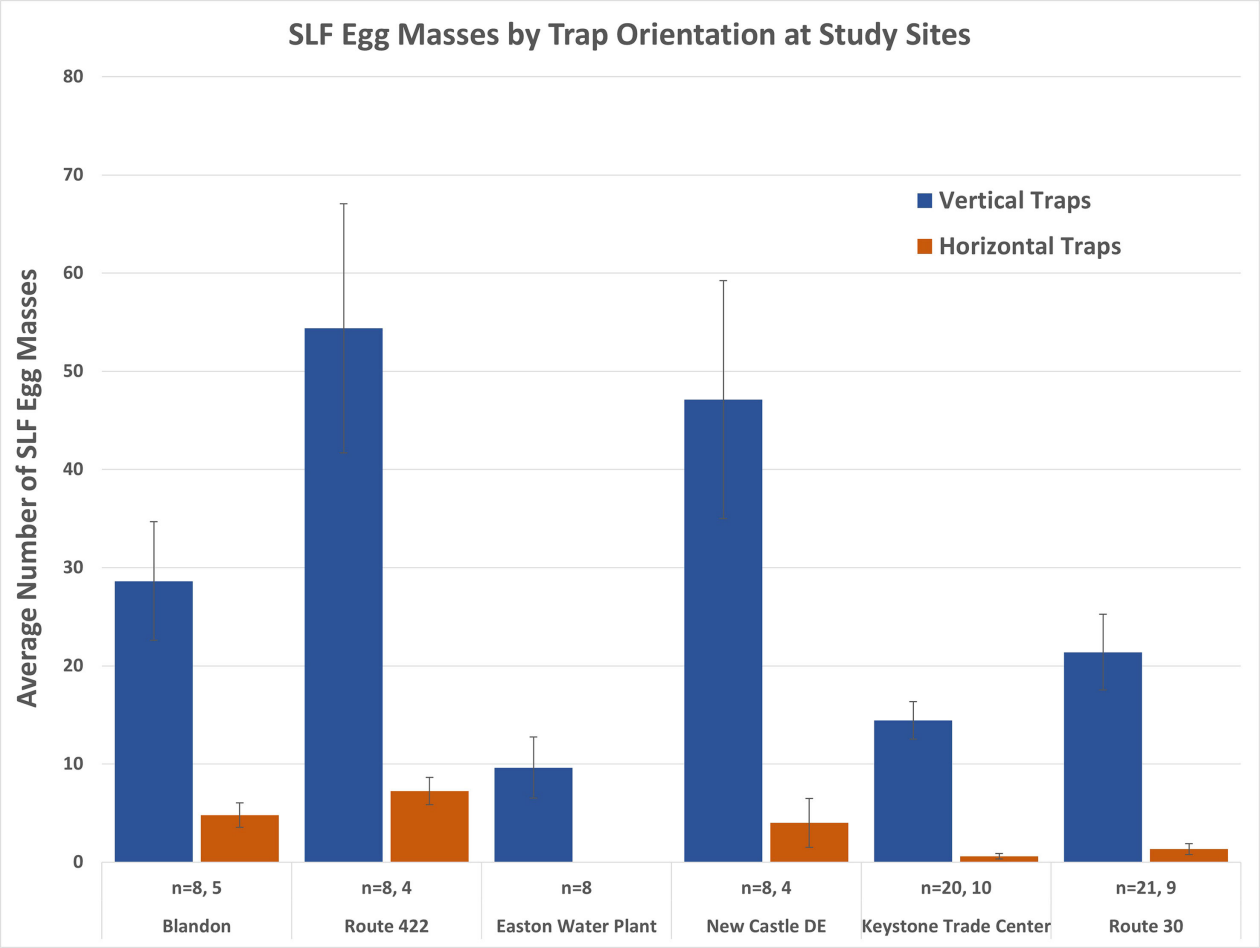
Average SLF egg masses laid in lamp shade traps oriented vertically and horizontally, by site. Vertically oriented traps were statistically different at each study site (range *P* < 0.027 to *P* < 0.001; Wilcoxon Rank Sum Test) except at the Easton site where no horizontal traps were set up.

Comparisons of the vertical and horizontal orientation of the traps for each study site were significantly different (range *P* < 0.027 to *P <* 0.001; Wilcoxon Rank Sum Test) for all comparisons (horizontal stems were not present at the Easton site). The average number of egg masses (± SE) per site for vertical and horizontal trap orientation are displayed in **Figure 9**. Horizontal trap catch averaged 0.6 to 7.3 egg masses per trap compared to 14.5 to 54.4 egg masses for the paired site comparisons. A vertical trap orientation increased trap catch by an average of 13.2 times (range 6-24) across study sites when using horizontal trap catch as a baseline. Trap catch on the horizontal traps was not statistically different from surveys of the number of egg masses present on horizontal surfaces to either side of the traps and on nearby control trees (Kruskal-Wallis ANOVA; F=0.99, df = 4,95, *P* = 0.38).

**Figure 10:**
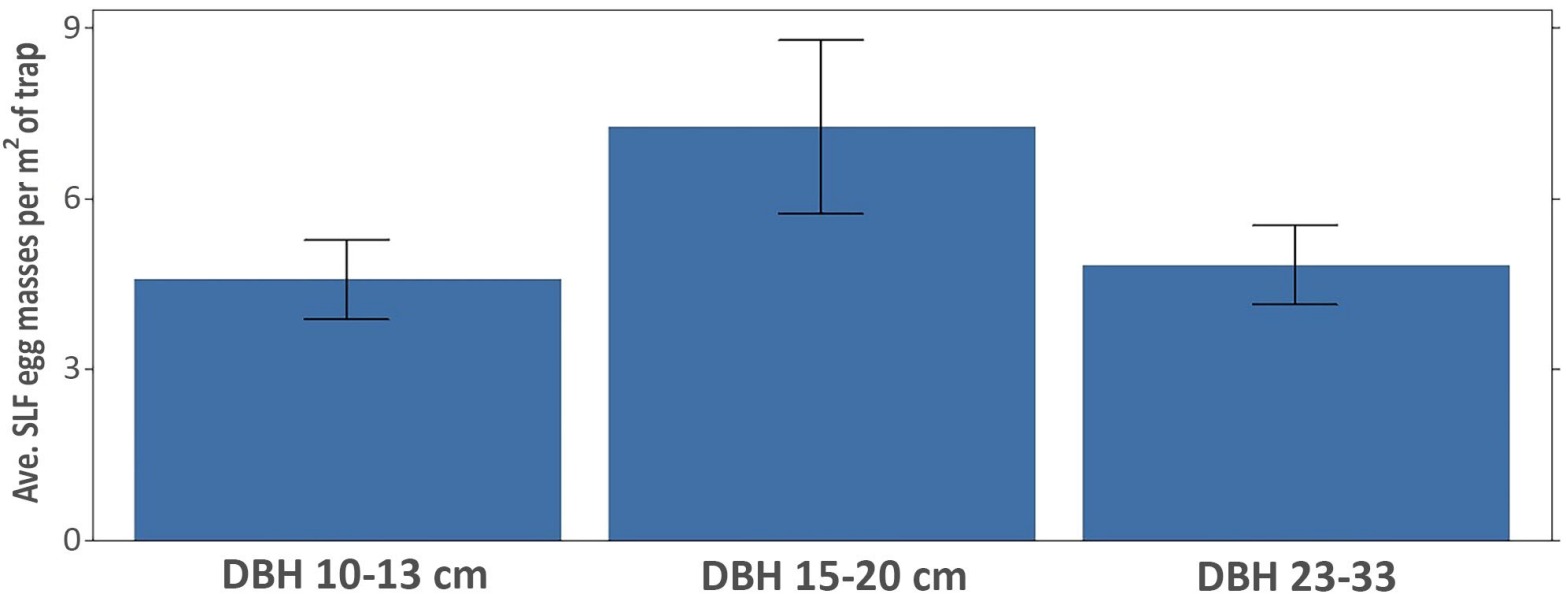
Average SLF egg masses (± SE) laid per m^2^ of trapping area on all vertically oriented traps, grouped by tree diameter. DBH classes were not statistically different (*P* = 0.66; Kruskal-Wallis ANOVA).

The vertical trap trees were grouped into three diameter classes as follows: 10-13 cm (n = 22); 15-20 cm (n = 26); 23-33 cm (n = 25). Traps on trees of the larger two size classes averaged (± SE) significantly more egg masses (29.3 ± 5.6 and 33.5 ± 4.9) than traps in the smallest size class (11.7 ± 1.6) (Kruskal-Wallis ANOVA; F = 7.66, df = 2,72, *P* < 0.001). However, when the number of egg masses per trap was adjusted for the diameter of the tree used for the trap and hence trap surface area, there is no statistical difference among the three diameter classes (**Figure 10**), although the 15-20 cm size group trended as being the most efficient for collecting SLF egg masses (Kruskal-Wallis ANOVA; F = 0.41, df = 2,72, *P* = 0.66).

## 4 Discussion

Our trapping data show that the LST is an effective oviposition trap for SLF egg masses. These traps are durable, low-cost, simple to construct and can be set up and left in the field until harvest. The trap is made of roofing material affixed around the trunk of the tree with a second layer of material inverted and held away from the tree such that the appearance is that of a lamp shade (**Figure 8**). A two-page document on how to construct the traps is provided in the supplementary materials. Lamp shade traps oriented vertically stimulated SLF females to focus oviposition on the trap substrate and very few egg masses were noted above or below the traps or on the trunks of the paired control trees. LSTs provide an environment and a material on which SLF females will greatly concentrate their egg masses.

Initial attempts at an SLF egg mass trap design began in 2018 following our field observations that egg mass placement did not seem to be totally random. SLF oviposition behavior resulted in egg masses being laid on a wide variety of objects, but large concentrations were also observed, often on the underside of limbs. Egg mass clusters also appeared to be laid in areas that stayed dry, so we incorporated a covered and uncovered design to test for this. We made incremental progress in designing a successful trap and deployed over 560 traps over four years before a significant number of egg masses were laid on a subset of the 2021 traps; traps that had a top or covering yielded four times more egg masses than open traps. However, covered traps averaged only 5.5 masses per trap, not sufficient to serve as a survey tool or practical for even collection of SLF eggs masses for research purposes. We decided to pursue this trapping effort an additional season, focusing on the suitable oviposition substrate we and other researchers had identified (roofing material) (personal communication: Dr. Leskey, USDA-ARS, Kearneysville, WV) using a single trap style that enhanced the protected environment SLF females respond to, which the 2021 testing had indicated.

Testing in 2021 had also shown that trap height placement did not impact trap catch, so traps in 2022 were placed at the convenient working height of about 1.4 m. To create a better environment, we considered that the triangle traps placed the oviposition substrate away from the tree trunk and SLF had to move off the tree to interact with it. For the 2022 trap design, we wrapped roofing material around and directly to the tree trunk and chose the LST design which funneled SLF into a sheltered environment as they moved up the tree. SLF nymphs and adults are very active and are readily caught in traps that take advantage of their propensity for positive upward movement (16). The success of this trap for SLF oviposition was that it combined the two factors we had identified into a single trap design. SLF females walk up and onto a suitable oviposition substrate, and then encounter an environment where oviposition behavior is stimulated.

It was unexpected that trap orientation was such a significant effect and that SLF females did not interact with the trap when placed horizontally. The vertical traps on living trees were compared to horizontal stems that had partially fallen and could accommodate a trap. Only 4.5% of the total egg masses in the traps were laid on horizontal traps, not statistically different from egg mass counts on either side of the horizontal traps and nearby control trees. Perhaps not as many SLF enter the horizontal traps or that this trap orientation fails to stimulate oviposition behaviors to the extent that a vertical trap orientation does. The number of egg masses found in horizontal traps reflected oviposition levels of SLF females in that immediate environment.

Vertical traps were deployed at each site on a range of tree diameters. This allowed us to identify the most efficient tree diameter on which to place LSTs, both in terms of efficiency (egg masses laid per trapping area) and for cost considerations of the amount of material required to build the traps. Although not statistically significant, traps placed on trees 15-20 cm in diameter averaged the most SLF egg masses per trapping area. Trees of this size can be selected for routine trapping of egg masses with the assurance that egg mass yield will be high and trapping materials can be kept to a minimum. LSTs can certainly be placed on larger diameter trees, but a greater effort and cost will result.

LSTs placed on trees other than TOH should still be attractive. Although we did not test other tree species, if SLF females are active and feeding on a different host tree (e.g., silver maple) and a trap is placed on that tree, there is no reason they would not interact with it as they do when the trap is placed on TOH. If a tree species has rough or uneven bark, we suggest first attaching a strip of batting material around the trunk at the bottom of where the LST will be installed. This will fill in any gaps between the bark and the first layer of roofing material, so SLF do not get under the trap as they travel upward. We did note a significant amount of mold present on the egg masses at most of the study sites. If egg masses are to be used for research purposes this can probably be mitigated by keeping rainwater from entering the top of the trap by stapling and draping a small tarp above and over the LST. There are also mold inhibitors that might help if sprayed up and into the trap every few weeks when wet and humid conditions are present.

LSTs are low-cost, easy to set up and take down and were very efficient at concentrating SLF egg masses. These traps have the potential to be a valuable tool not only to aid in the collection of egg masses that are needed for the active biological control and research efforts against this invasive, destructive insect but also as a trapping tool that can be used to accumulate and destroy egg masses while monitoring SLF populations in a woodlot. This trap has great potential for detecting SLF in new areas, for monitoring SLF in areas of concern and potentially for estimating and predicting SLF population levels in an infested area.

## Data availability statement

The raw data supporting the conclusions of this article will be made available by the authors, without undue reservation.

## Author contributions

PL and AD-F conceived the project. PL acquired funding. All authors participated in the design of the experiments. EW and AD-F oversaw the experiments, coordinated and participated in field work including trap set up, sampling and data collection and summary. PL conducted data analysis. PL drafted the manuscript with contributions from AD-F and EW. All authors contributed to the article and approved the submitted version.
