# Supplementary material for: An effective trap for spotted lanternfly egg masses
Source: Front Insect Sci. 2023 Apr 17;3:1154510. doi: 10.3389/finsc.2023.1154510 (PMC10926488; doi:10.3389/finsc.2023.1154510)
Supplement: Supplementary file 1 [file DataSheet_1.pdf]

Lampshade Trap Construction for Spotted Lanternfly Egg Masses

Materials Needed (supplied links are suggestions and do not imply endorsement of products or companies by USDA):

- Roll of Peel and Stick Roofing Material 9" x 33'
- Zip ties (18" or longer, reusable option here)
- Box cutter, DBH tape, staple gun (½"staples)
- Batting material 3.5" wide (thin cotton alternative)

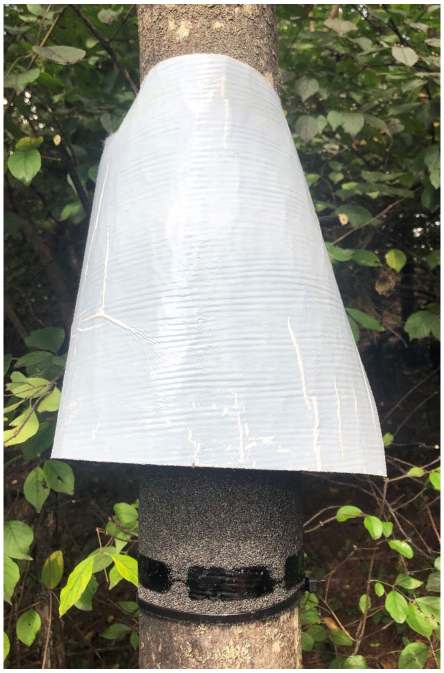

Background

Our research group has been investigating SLF egg laying behavior for several years. After much trial and error, we identified an environment that encourages SLF females to lay eggs within a trap. The trapping material is flexible and allows egg masses to be collected and/or counted and disposed of. The trap is efficient, low-cost, and easy to set up.

Methods

Suitable sites and trees

If egg masses are to be collected, you will want to select areas with medium to high SLF populations. Tree of Heaven (TOH, *Ailanthus altissima*) were used to develop these traps. However, the traps can be placed on other species of trees that are infested. Ideally, trees used for trapping should be 6 to 8" in DBH. Smaller (< 4 inch DBH) and larger trees (> 8" DBH) are less productive per ft^2 of trap area. Trap height should be at approximately 3.5 to 5 ft high on the tree for convenience. Avoid bark irregularities or wounds.

Installation ***Note: DO NOT peel the white plastic backing off the roll of roofing material to expose the adhesive. The plastic backing helps repel moisture and makes it easier to harvest egg masses, if desired. It is most efficient to have 2 people setting up a trap.**

1. Determine the length of roofing material needed by wrapping the roll of material flush around the tree at the desired height. The textured side of the roofing material should face outwards. Allow the material to slightly overlap itself by about 1" and make a notch in the material with a box cutter so you know where to cut. Remove the roofing material, lay it on the ground, then use a box cutter to cut the material as straight as possible.

   Take the cut piece of roofing material and wrap it tight and flush against the tree with the material slightly overlapping itself. The textured asphalt side will face outwards. Use a staple gun and staple the material to the tree where the material overlaps (A). Usually about 4 staples will be sufficient. Take a long zip-tie (or several zip-ties in series) and place it around the very bottom of the material. Cinch up the zip tie tightly to secure the material to the tree (B). You can cut off any excess zip-tie material.

2. To measure the length of the batting material that will be needed, wrap the 3.5" wide batting material flush against the tree trunk, right above the roofing material, so that is slightly overlaps itself. To attach the batting to the tree, fold the piece of batting material in half upwards and wrap it around the tree trunk (C). Secure it with a zip-tie (or several zip-ties in series) wrapped around the middle of the batting material (D). Cut excess zip-tie tail if it's longer than about 3".

3. Measure the length of roofing material needed for the outer portion of the trap (which looks like a lampshade). **This layer will have the textured, black side facing inwards**. To do this, wrap the roofing material around the tree just above the height of the batting material, and this time, use enough roofing material to create a 2 ½” overlap. There should also be enough material so that the bottom is flared out about 2” from the tree (E). This creates enough space for SLFs to crawl up and inside the trap. After the material is measured so there is enough for a 2” gap, use a box cutter to cut the material needed.

Take the cut piece of “lampshade” roofing material facing *inwards*. Wrap it around the tree with the top about 1” above the batting material so that it covers the portion of the roofing material already secured to the tree. Adjust the bottom of the lampshade so there is a continuous gap about 2” away from the tree, overlapping the lampshade material around itself. Use a staple gun to staple the lampshade to the tree right above the batting material, making sure to retain the gap between the bottom edge of the material and the tree (F). <u>You may need to pull on the lampshade portion and reposition it as you staple</u>. Start with roughly 3” between each staple. Use as many staples as necessary to secure the lampshade to the tree while still maintaining the 2” bottom gap between materials. If needed, staple the lampshade ends together at the bottom to minimize flaring. There will be ridges at the top of the lampshade due to the flaring shape. Take small pieces of batting material and fill in the gaps of the ridges on top (G). <u>Do not remove the plastic backing from the roofing material (E-G).</u>

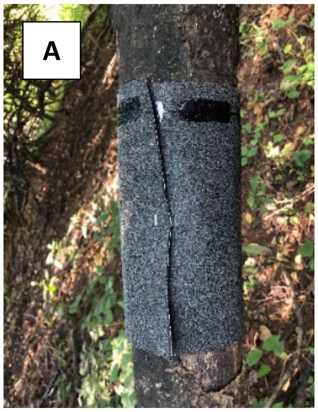

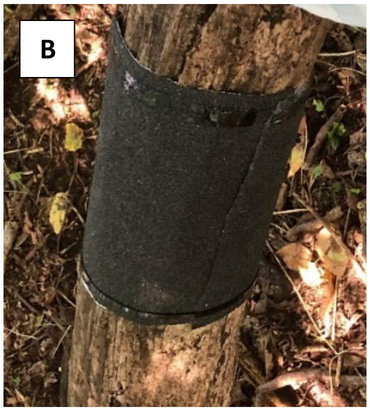

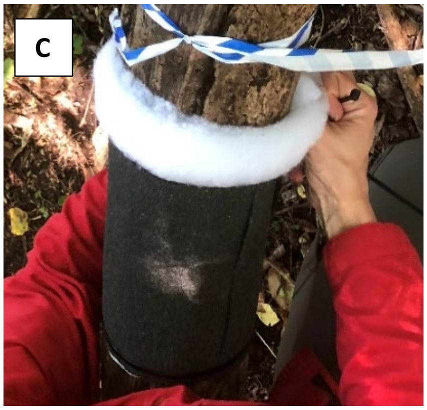

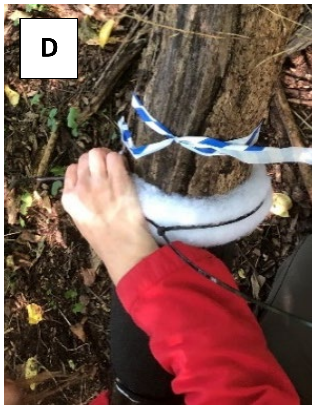

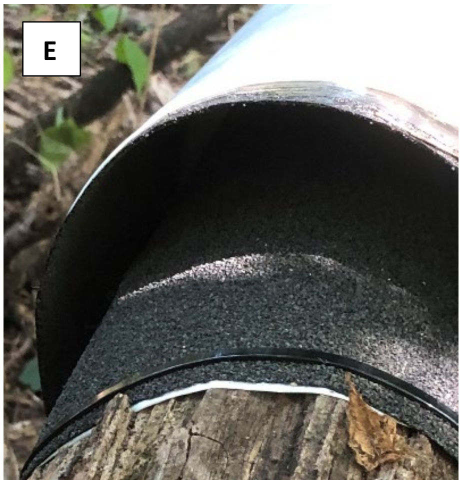

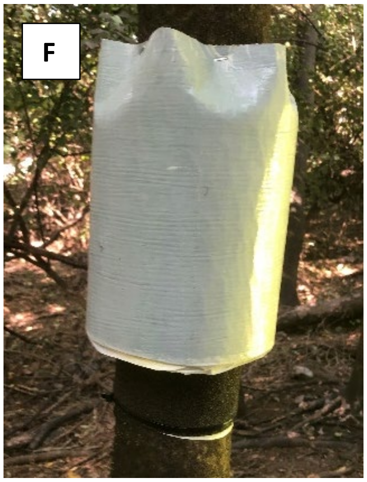

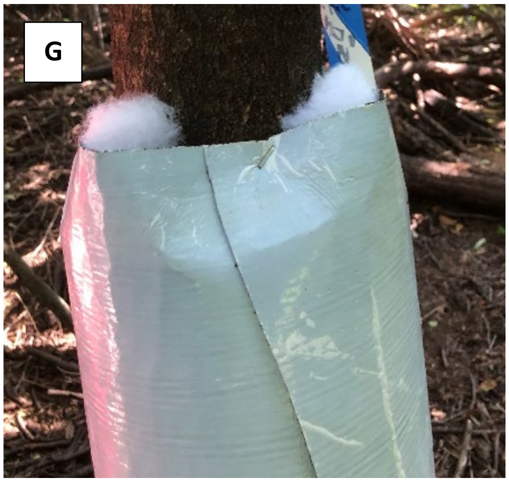

Point of Contact

<u>Phillip Lewis:</u>
USDA-APHIS Forest Pest Methods Laboratory
1398 W Truck Road
Buzzards Bay, MA 02542
Phillip.A.Lewis@USDA.GOV 508-563-0914
